# Dependence of α-helical and β-sheet amino acid propensities on the overall protein fold type

**DOI:** 10.1186/1472-6807-12-18

**Published:** 2012-08-02

**Authors:** Kazuo Fujiwara, Hiromi Toda, Masamichi Ikeguchi

**Affiliations:** 1 Department of Bioinformatics, Soka University, 1-236 Tangi-cho, Hachioji, Tokyo 192-8577, Japan

## Abstract

**Background:**

A large number of studies have been carried out to obtain amino acid propensities for α-helices and β-sheets. The obtained propensities for α-helices are consistent with each other, and the pair-wise correlation coefficient is frequently high. On the other hand, the β-sheet propensities obtained by several studies differed significantly, indicating that the context significantly affects β-sheet propensity.

**Results:**

We calculated amino acid propensities for α-helices and β-sheets for 39 and 24 protein folds, respectively, and addressed whether they correlate with the fold. The propensities were also calculated for exposed and buried sites, respectively. Results showed that α-helix propensities do not differ significantly by fold, but β-sheet propensities are diverse and depend on the fold. The propensities calculated for exposed sites and buried sites are similar for α-helix, but such is not the case for the β-sheet propensities. We also found some fold dependence on amino acid frequency in β-strands. Folds with a high Ser, Thr and Asn content at exposed sites in β-strands tend to have a low Leu, Ile, Glu, Lys and Arg content (correlation coefficient = −0.90) and to have flat β-sheets. At buried sites in β-strands, the content of Tyr, Trp, Gln and Ser correlates negatively with the content of Val, Ile and Leu (correlation coefficient = −0.93). "All-β" proteins tend to have a higher content of Tyr, Trp, Gln and Ser, whereas "α/β" proteins tend to have a higher content of Val, Ile and Leu.

**Conclusions:**

The α-helix propensities are similar for all folds and for exposed and buried residues. However, β-sheet propensities calculated for exposed residues differ from those for buried residues, indicating that the exposed-residue fraction is one of the major factors governing amino acid composition in β-strands. Furthermore, the correlations we detected suggest that amino acid composition is related to folding properties such as the twist of a β-strand or association between two β sheets.

## Background

In 1974, Chou and Fasman published the calculated frequency of occurrence and conformational propensity of each amino acid in the secondary structures of 15 proteins, consisting of 2473 amino acid residues [1]. Since then, a vast number of protein structures have been determined and classified to reflect both structural and evolutionary relatedness [2,3]. SCOP classification (Structural Classification of Protein) is one of the major database which provides a detailed and comprehensive description of the relationships of all known proteins structures. The classification is on hierarchical levels: the first two levels, family and superfamily, describe near and far evolutionary relationships; the third, fold, describes geometrical relationships. Most of the folds (899/1086) are assigned to one of the four structural classes; “all-α”, “all-β”, “α/β” (for proteins with α-helices and β-strands that are largely interspersed) and “α + β” (for those in which α-helices and β-strands are largely segregated). Remaining folds are assigned to "Multi-domain", "Membrane and cell surface" or "Small" proteins classes. In 2009, we developed a quaternary structural database for proteins, OLIGAMI [4] in which the oligomer information was added to the SCOP classification [2], to allow an exhaustive survey of tertiary or quaternary structures of proteins.

A large number of studies have been carried out to obtain amino acid propensities for α-helix and β-sheet [1,5-28]. The propensities have been estimated from statistical analysis of three-dimensional structures [1,6-15], experimental determination of α-helix or β-sheet content in peptides [16-23], and experimental determination of the thermodynamic stability of mutant proteins [23-28]. The obtained propensities for α-helix are consistent between studies, with the pair-wise correlation coefficient (R) frequently being >0.8, although Richardson et al. [7] and Engel et al. [12] showed that amino acid propensities are different for specific locations of α-helix depending on amino acids. Engel et al. also show that most helices are amphiphilic and have a strong tendency to both begin and end on the solvent-inaccessible face of the α-helix, suggesting that the propensities for α-helix differ between solvent-accessible and solvent-inaccessible faces. On the other hand, the β-sheet propensities obtained by several studies differ significantly, indicating that the context significantly affects β-sheet propensity. β-sheets consist of various combination of β-strands; the number of strands, parallel, anti-parallel, mixed β-sheet and so on. For IgG-binding domain from protein G, which have four antiparallel β-strands, Minor and Kim showed that β-sheet propensity measured at the center strand [27] differs significantly from that measured at an edge strand [28]. This context-dependent nature of the β-sheet propensity may be reflected in its dependence on overall protein fold. Previously, Jiang et al. [10] and Costantini et al. [13] calculated the secondary structure propensities for four protein structural classes; “all-α”, “all-β”, “α/β”, and “α + β” and showed that β-sheet propensity depends on these structural classes. However, it has not been clarified that their dependencies result from the difference in what kind of context, since each folding class contains various folds that have different context. So it is interesting to address whether the amino acid propensity of each amino acid vary depending on the fold type.

In this study, to clarify the relationship between the amino acid propensity and the context in more detail, we calculated the occurrence of each amino acid residue in α-helical and β-strand conformations as a function of the SCOP fold of the protein (*i.e.* lower structural level than previously addressed), and categorized the residues as exposed to solvent or buried interior. The results indicate that α-helix propensities do not differ significantly by fold but that β-sheet propensities are diverse and indeed depend on the fold. Furthermore, we found the some relationships between a structural feature and an amino acid composition by analyzing correlations between a protein fold and an amino acid propensity.

## Methods

### Selecting protein structures to be included in the dataset

This study uses sets of non-redundant PDB entries (three-dimensional coordinates) in each fold type. To facilitate the analysis, we wanted to extract monomeric or homo-oligomeric and single-domain proteins from PDB. This has been accomplished in OLIGAMI (http://protein.t.soka.ac.jp/oligami/) [4] which is database combined SCOP database (Structural Classification of Proteins) [2] and oligomeric information. From these coordinates, a non-redundant subset of PDB entries (in which no pair of structures had >60% sequence identity) was created for each fold of the four main SCOP classes of proteins: “all-α”, “all-β”, “α/β”, and “α + β”. The number of proteins (or protein domains) classified in each SCOP fold varies; for example, the SCOP fold “dipeptide transport proteins” contains only one entry, that of d-amino peptidase (PDB, 1HI9). This enzyme is a decamer of identical subunits, each with 88 and 68 residues in α-helical and β-strand conformations, respectively. Because the number of residues in this SCOP fold category is too small to extract statistically meaningful results, we selected only those SCOP folds that contained at least 2,000 residues in an α-helical, β-strand, or other conformation (Table 1). Consequently, we identified 39 (2,029 PDB entries) of 899 SCOP folds for the dataset of α-helices and 24 (1,879 PDB entries) of 899 SCOP folds for the dataset of β-strands. Twelve of these SCOP folds, such as the TIM barrel and Rossmann fold—both examples of α/β proteins—were included in the dataset for both α-helices and β-strands, and consequently we used 51 SCOP folds. We also identified 39 of 51folds for the dataset of other conformation as a control. SCOP release 1.73 was used for all calculations. 

**Table 1:** SCOP folds included in the dataset of α-helices, β-strands and other conformation

**SCOP Class**	**SCOP Fold**	***NE***_***j***_^**1**^	***N***_***j***_^**2**^	**α-helix**		**β-strand**		**Other**	
				***N***^***α***^_***j***_^**3**^**(*f***^***αexp***^_***j***_**)**^**6**^		***N***^***β***^_***j***_^**4**^**(*f***^***βexp***^_***j***_**)**^**7**^		***N***^***O***^_***j***_^**5**^**(*f***^***Oexp***^_***j***_**)**^**8**^	
All α proteins	4-helical cytokines	24	3,452	2,272	(50)				
	Alpha/alpha toroid	25	10,766	5,410	(32)			4,611	(56)
	Alpha-alpha superhelix	48	11,524	8,213	(48)			3,270	(76)
	Cytochrome P450	19	7,811	4,042	(43)			3,008	(63)
	EF Hand-like	56	7,106	4,244	(54)			2,739	(76)
	Ferritin-like	40	7,261	5,354	(35)				
	Four-helical up-and-down bundle	45	5,800	4,344	(55)				
	Globin-like	30	4,223	3,161	(56)				
	HD-domain/PDEase-like	12	3,487	2,248	(43)				
	Heme oxygenase-like	15	3,364	2,369	(43)				
	L-aspartase-like	14	6,699	4,154	(32)			2,217	(45)
	Nuclear receptor ligand-binding domain	20	4,747	3,221	(48)				
α/β proteins	Adenine nucleotide alpha hydrolase-like	33	7,219	3,398	(49)			2,716	(60)
	ALDH-like	11	5,122	2,198	(43)				
	Alpha/beta-Hydrolases	74	23,468	9,192	(42)	3,985	(23)	10,291	(54)
	ClpP/crotonase	19	4,736	2,369	(38)				
	Flavodoxin-like	96	17,354	7,063	(49)	3,218	(24)	7,073	(58)
	HAD-like	47	10,900	4,853	(54)			4,231	(59)
	Isocitrate/Isopropylmalate dehydrogenase-like	17	6,267	2,869	(45)			2,239	(56)
	NAD(P)-binding Rossmann-fold domains	100	26,500	12,116	(40)	4,312	(18)	10,072	(57)
	Nucleotide-diphospho-sugar transferases	24	6,359	2,297	(47)			2,723	(61)
	Periplasmic binding protein-like II	46	14,225	5,593	(49)	2,916	(29)	5,716	(59)
	Phosphorylase/hydrolase-like	36	9,424	3,277	(43)	2,105	(22)	4,042	(57)
	P-loop containing nucleoside triphosphate hydrolases	170	39,025	16,859	(52)	7,524	(27)	14,882	(64)
	PLP-dependent transferases	77	30,112	13,025	(43)	4,509	(16)	12,578	(43)
	Restriction endonuclease-like	30	6,407	2,631	(50)			2,447	(64)
	Ribokinase-like	24	7,234	3,029	(42)			2,589	(59)
	Ribonuclease H-like motif	33	6,808	2,832	(49)			2,585	(70)
	S-adenosyl-L-methionine-dependent methyltransferases	92	23,315	8,567	(49)	5,538	(31)	9,210	(62)
	SIS domain	14	4,150	2,171	(38)				
	Thioredoxin fold	82	11,085	3,845	(59)	2,400	(29)	4,840	(67)
	TIM beta/alpha-barrel	261	81,525	35,904	(46)	12,411	(11)	33,210	(53)
	Tryptophan synthase beta subunit-like PLP-dependent enzymes	18	6,296	2,821	(36)			2,503	(53)
	UDP-Glycosyltransferase/glycogen phosphorylase	16	6,976	3,324	(44)			2,562	(56)
α + β proteins	Acyl-CoA N-acyltransferases (Nat)	63	11,104	3,872	(57)	3,376	(33)	3,856	(74)
	Cysteine proteinases	34	9,122	3,027	(44)			4,248	(64)
	Ferredoxin-like	174	19,761	6,048	(57)	5,212	(36)	8,501	(73)
	Protein kinase-like (PK-like)	57	16,585	6,566	(44)	2,783	(40)	7,236	(65)
	Thioesterase/thiol ester dehydrase-isomerase	38	5,319			2,090	(43)		
	Zincin-like	33	9,786	4,555	(44)			4,099	(63)
All β proteins	6-bladed beta-propeller	18	6,195			2,785	(23)	3,083	(60)
	Concanavalin A-like lectins/glucanases	60	13,290			6,242	(32)	6,122	(67)
	Double-stranded beta-helix	68	15,315			5,425	(27)	7,075	(62)
	Galactose-binding domain-like	29	4,522			2,191	(39)	2,077	(71)
	Immunoglobulin-like beta-sandwich	117	13,954			6,176	(47)	7,225	(78)
	Lipocalins	41	6,422			3,120	(45)	2,371	(75)
	OB-fold	58	7,072			2,665	(41)	3,418	(75)
	PH domain-like barrel	54	7,041			2,434	(42)	3,393	(83)
	Single-stranded right-handed beta-helix	27	8,817			3,600	(31)	4,484	(59)
	Trypsin-like serine proteases	41	9,275			3,290	(29)	4,827	(63)

1. *NE *_*j*_ : The number of PDB entries in the fold, j.

2. *N *_*j*_ : The number of residues in the fold, j.

3. *N *^*α*^_*j*_ : The number of residues of α-helices in the fold, j.

4. *N *^*β*^_*j*_ : The number of residues of β-strands in the fold, j.

5. *N *^*O*^_*j*_ : The number of residues of other conformation in the fold, j.

6. *f *^*αexp*^_*j*_ : Fraction (%) of exposed residues in α-helices in the fold, j.

7. *f *^*βexp*^_*j*_ : Fraction (%) of exposed residues in β-strands in the fold, j.

8. *f *^*Oexp*^_*j*_ : Fraction (%) of exposed residues in other conformation in the fold, j.

### Determining amino acid propensities in the secondary structure elements

The propensity, *P*_*ij*_, of amino acid, *i*, for SCOP fold, *j*, in α-helices (*P*^*α*^_*ij*_) or β-strands (*P*^*β*^_*ij*_) was calculated as follows:

(1) $$P_{\mathrm{ij}}^{S}=\frac{f_{\mathrm{ij}}^{S}}{f_{i}}$$

where *f*^*S*^_*ij*_ is the frequency of the amino acid *i* occurring in SCOP fold *j* in the secondary structure *S* (*f*^*S*^_*ij*_ = *N*^*S*^_*ij*_/*N*^*S*^_*j*_), and *f*_*i*_ is the frequency of the amino acid *i* occurring in the protein (*f*_*i*_ = *N*_*i*_/*N*_*t*_). *N*^*S*^_*ij*_ and *N*^*S*^_*j*_ are the number of amino acid *i*, and the number of all amino acids in the secondary structure *S* in SCOP fold *j*. *N*_*i*_ is the number of amino acid *i*, and *N*_*t*_ is the total number of amino acids in all 51 SCOP folds. Therefore, the propensity means a relative quantity of the frequency of the amino acid *i* occurring in a secondary structure in a specific fold divided by the frequency of the amino acid *i* occurring in all proteins. If *P*^*α*^_*i*_ = 1, the amino acid *i* is contained equally in both the α-helical region and the protein. When *P*^*α*^_*i*_ > 1, the amino acid *i* is more frequent in the α-helical region than in the protein. The standard deviation for the normalized function, *P*_*ij*_, was calculated as follows.

(2) $$\sigma_{P_{\mathrm{ij}}}=\sqrt{\frac{f_{\mathrm{ij}}\left(1-f_{\mathrm{ij}}\right)}{N_{j}}\cdot \frac{N_{t}}{N_{i}}}$$

The secondary structure assignment program DSSP [29] was used for all secondary structure assignments. DSSP program assigns secondary structures, i.e., H: α-helix, G: 3_10_-helix, I: 5-helix (π-helix), E: extended strand, B: residue in isolated β-bridge, S: bend and T, hydrogen bonded turn. We regarded H: α-helix and G: 3_10_-helix as α-helix and E as β-strand, and remaining residues except T are defined as other conformation.

### Defining exposed and buried residues in the secondary structure elements

Amino acid residues were defined as “exposed” when >20% of the total accessible surface area was exposed to solvent. This threshold level of 20% was determined as the value that could classify an almost equal number of residues as exposed (1,241 residues) or buried (1,276 residues) in β-strands for 37 soluble β-barrel proteins. The total accessible surface area for a given amino acid, X, was calculated using the tri-peptide (G-X-G), using DSSP [29]. The frequency of exposed, *f*^*Sexp*^_*ij*_, and buried, *f*^*Sbur*^_*ij*_, residues was calculated for each amino acid in an α-helical or β-strand conformation for each SCOP fold. The propensities for an α-helical or β-strand conformation for each SCOP fold for exposed residues, *P*^*Sexp*^_*ij*_, and buried residues, *P*^*Sbur*^_*ij*_, were obtained by dividing *f*^*Sexp*^_*ij*_ and *f*^*Sbur*^_*ij*_ by the frequency of the exposed and buried residues in all SCOP folds, *f*^*exp*^_*i*_ and *f*^*bur*^_*i*_, respectively,

(3) $$P_{\mathrm{ij}}^{S\text{exp}}=\frac{f_{\mathrm{ij}}^{S\text{exp}}}{f_{i}^{\text{exp}}}$$

(4) $$P_{\mathrm{ij}}^{\text{Sbur}}=\frac{f_{\mathrm{ij}}^{\text{Sbur}}}{f_{i}^{\text{bur}}}$$

### Population difference test

The Fisher-Irwin population test can be used to determine statistically significant differences between *P*_*ij*_ values for different fold types. Because the *n* value (the sum of the number of each amino acid, *i*, from both populations) was large, the exact Fisher-Irwin test values were not calculated. Instead, a large sample number approximation was used [30].

(5) $$Z=\frac{f_{i1}-f_{i2}}{\sqrt{\frac{f_{i1}\left(1-f_{i1}\right)}{N_{i1}}+\frac{f_{i2}\left(1-f_{i2}\right)}{N_{i2}}}}$$

The *P*_*ij*_ value difference between the populations is considered significant if the test variable Z is >1.25, which corresponds to a 90% confidence level, then the populations were considered to be different.

## Results and discussion

### Amino acid propensities for the α-helical or β-strand conformation

For individual amino acids, a *P*^*α*^ of <0.9 denotes an α-helix breaker, a *P*^*α*^ of >1.1 denotes an α-helix-favored amino acid, and values between 0.9 and 1.1 denote that the amino acid is neutral in this regard [31]. The same principle applies to *P*^*β*^. The amino acid propensities calculated using our dataset (*P*^*α*^_*i*_ and *P*^*β*^_*i*_ ) are shown in Table 2. Their standard deviations ranged from 0.001 to 0.004. The results are in good agreement with previous reports [1,6,10]. 

**Table 2:** Mean amino acid propensities for α-helix and β-strand conformations

**Amino acid**	**α-helix**			**β-strand**		
	**Exposed residues**	**Buried residues**	**Total residues**	**Exposed residues**	**Buried residues**	**Total residues**
V	0.83	0.89	0.91	2.31	1.57	2.00
I	0.96	1.01	1.04	2.02	1.39	1.79
L	1.16	1.27	1.28	1.18	0.93	1.15
M	1.03	1.29	1.26	1.01	0.84	1.01
P	0.48	0.41	0.44	0.49	0.42	0.40
A	1.43	1.37	1.41	0.48	0.72	0.75
C	0.63	0.85	0.85	1.24	1.07	1.36
F	0.88	0.99	1.00	1.50	1.10	1.4
Y	0.91	0.98	0.98	1.71	1.12	1.37
W	0.87	1.09	1.07	1.90	0.91	1.23
Q	1.34	1.21	1.26	0.96	0.82	0.72
S	0.74	0.80	0.76	0.86	0.85	0.81
T	0.72	0.84	0.78	1.58	1.08	1.21
N	0.74	0.77	0.73	0.71	0.76	0.63
H	0.90	0.85	0.87	1.15	0.98	0.99
D	0.91	0.73	0.82	0.61	0.76	0.55
K	1.25	1.13	1.17	1.14	0.98	0.76
E	1.51	1.25	1.39	0.89	0.86	0.65
R	1.31	1.13	1.21	1.27	0.82	0.85
G	0.28	0.59	0.44	0.41	0.81	0.67

We also calculated the amino acid propensities for exposed and buried residues (*P*^*exp*^_*i*_ and *P*^*bur*^_*i*_) in the secondary structural elements (Table 2). For α-helices, the three mean propensities *P*^*α*^_*i*_, *P*^*αexp*^_*i*_ and *P*^*αbur*^_*i*_ have similar trends. On the other hand, mean propensities for exposed residues (*P*^*βexp*^_*i*_) and buried residues (*P*^*βbur*^_*i*_) for β-strands differ significantly (Table 2). It is especially interesting that Lys and Arg, but not two other charged residues, Asp and Glu, are preferred as exposed residues in β-strands. Not surprisingly, all charged amino acids are disfavored as buried residues in β-strands. The buried regions disfavor charged amino acids for β-strands, whereas the α-helix can tolerate charged amino acids.

As previously reported in statistical studies, charged amino acids (including Lys and Arg) yield low values for *P*^*β*^[1,6,10,13], which is in agreement with the mean propensities, *P*^*β*^_*i*_, determined in the present work. Our results, however, show that Lys and Arg have relatively high *P*^*βexp*^ values for exposed residues, but this property is masked when comparing mean propensities. In our dataset, the fraction of exposed residues in β-strands is low (29%) compared to α-helices (46%). Most residues in β-strands are buried inside proteins and covered by α-helices or loop regions; exposed residues are thus less frequently encountered in β-strands, and their contributions to the mean *P*^*β*^_*i*_ are therefore small. Jiang and coworkers [10] have suggested that the hydrophobicities of amino acid side chains are the key determinant of β-sheet structures, but our data suggest that this result is true for buried residues but not for exposed residues in β-sheet structures. Minor and Kim [27] measured the propensity of the 20 amino acids for the β-sheet formation in a variant of the IgG-binding domain from protein G, which have four antiparallel β-strands. Amino acid substitutions were made at a guest site on the solvent-exposed surface of the center strand. The propensities from those experiments show a strong correlation with the logarithmic *P*^*βexp*^_*i*_ values obtained here (R = 0.82), although they show a weaker correlation with our logarithmic *P*^*βbur*^_*i*_ values (R = 0.63). Furthermore, there is poor correlation between the propensities determined by Minor and Kim [27] and those of Chou and Fasman [1]. These results show that the preference for β-strands differs for exposed and buried sites.

### Fold dependency of amino acid propensities for α-helices

**Figure 1:**
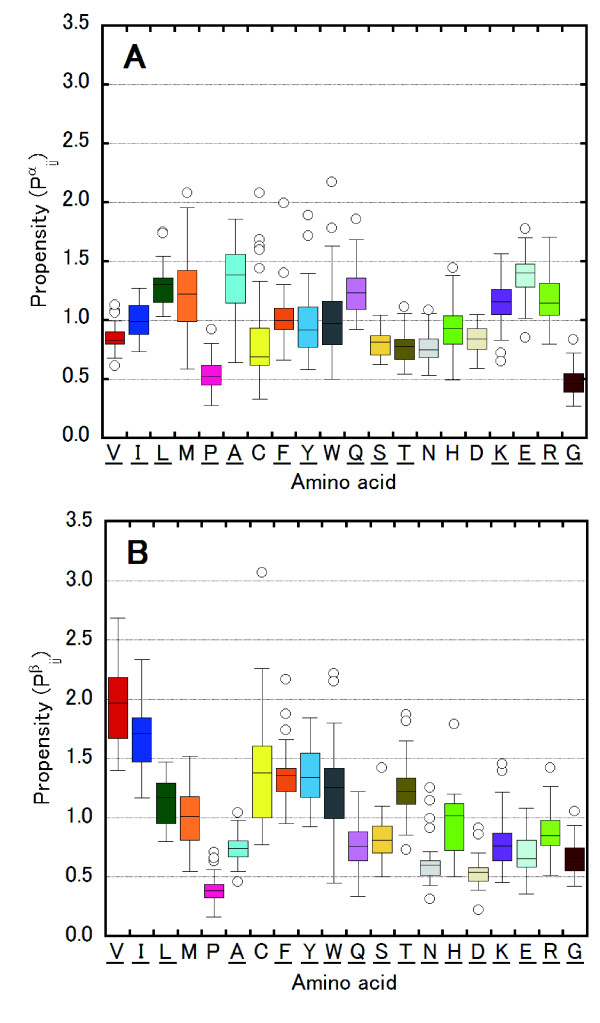
**Amino acid propensities for each SCOP fold. **Box plots of amino acid propensities for each SCOP fold for α-helices (**A**) and β-strands (**B**). Each box encloses 50% of the data with the median value displayed as a line. The top and bottom of the box mark the limits of ±25% of the data. The lines extending from the top and bottom of each box mark the minimum and maximum values within the data set that fall within an acceptable range. Any value outside of this range, called an outlier, is displayed as an individual point. Underlining of certain residues (one-letter code) on the horizontal axis denotes that the results from the Fisher-Irwin population proportion test indicated that differences in propensities are statistically significant between folds.

The propensities of amino acid *i* in the helical region of fold *j*, *P*^*α*^_*ij*_, and the β-strand region of fold *j*, *P*^*β*^_*ij*_, were thus calculated for 39 and 24 of SCOP folds, respectively (Figure 1). Their standard deviations range from 0.01 to 0.05. With the exception of Met, Cys, Trp, Asn, Asp and His for *P*^*α*^_*ij*_, and with the exception of Met, Pro and Cys for *P*^*β*^_*ij*_, the population of amino acids differed (>90% confidence level) for more than one pair of folds.

In particular, a wide range of *P*^*α*^_*ij*_ values was obtained for the aromatic residues Phe (0.66–2.00) and Tyr (0.58–1.89), depending on fold type, and the mean propensity for all folds is approximately 1.0 for these amino acids (Figure 1A and Table 2). The propensities of the charged residues Lys (0.65–1.56) and Arg (0.80–1.71) also varied widely depending on a fold. On the other hand, in >80% of SCOP folds, Leu or Glu are favored in the α-helical conformation, whereas Val, Pro, Ser, Thr, Asn, Asp and Gly are disfavored. Ala is favored in the α-helical conformation in the majority of the folds (79%) but is disfavored in two folds (Protein kinase-like and 4-helical cytokines). In particular, the value of the propensity of Ala for the "4-helical cytokines" fold is quite low (*P*^*α*^_*ij*_ = 0.64). Met, Cys, Trp and His do not have a fold-type population difference at the >90% confidence level in any pair of folds, although their propensities vary widely among the various folds. Therefore, we did not further assess these amino acids.

Richardson et al. showed that Ala is not favored in ends of α-helix [7], suggesting that a short α-helix does not favor Ala. The mean length of α-helix of the 4 helical cytokines fold is, however, the third longest of those of 39 folds (The longest and the second longest are those of "Ferritin-like" and "Four-helical up-and-down bundle" folds, respectively). Then, the correlation coefficient between the mean length of α-helix and the amino acid propensity for each amino acid were calculated, so that they were smaller than 0.4. This result indicates that there is no relationship between the mean length of α-helix and the helical propensity of any amino acid.

Engel et al. show that most helices are amphiphilic [7,12], suggesting that the propensities for α-helix depend on the exposed residue fraction. So, we examined the correlations between the exposed residue fraction and the frequency of amino acids in α-helices. No amino acid showed a strong correlation (R < −0.7 or R > 0.7) between the exposed residue fraction and the amino acid frequency, although the charged residues, Lys and Asp have a relatively strong positive correlation (R_K_ = 0.66, R_D_ = 0.54). In contrast, the correlation coefficients of Glu and Arg (also charged amino acids) are small (R_E_ = 0.26, R_R_ = 0.07).

**Figure 2:**
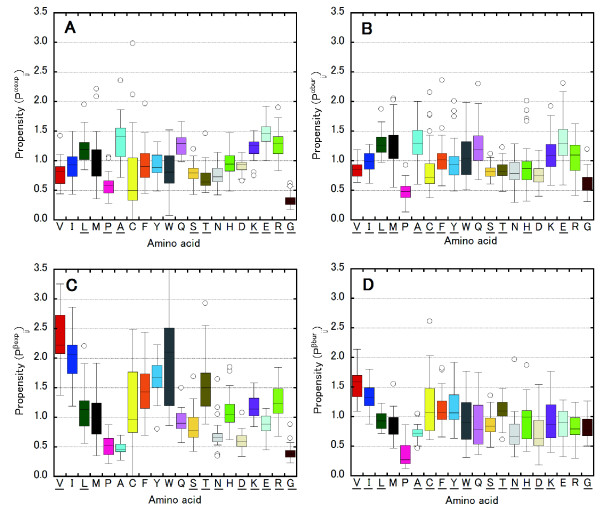
**Amino acid propensities for exposed and buried residues. **Box plots of Amino acid propensities for each SCOP fold for exposed (**A**) and buried (**B**) residues in α-helices and for exposed (**C**) and buried (**D**) residues in β-strands. The propensities for β-strands for Trp in the “PH domain-like barrel” SCOP fold and for Lys in the “Protein kinase-like” SCOP fold were out of range (4.3 in **C** and 3.8 in **D**, respectively) and are not shown. Underlining of certain residues on the horizontal axis denotes that the results from the Fisher-Irwin population proportion test indicated that differences in propensities are statistically significant between folds.

Figure 2 also presents propensities for exposed and buried amino acids for each SCOP fold. For the exposed regions of an α-helix (Figure 2A), less than ten amino acids show the population difference with 90% confidence for at least one pair of folds. Probably, this results from the fact that the dataset was limited to exposed residues. Glu (*P*^*αexp*^_*ij*_: 1.0–1.92) is favored in exposed regions (Figure 2A) whereas Leu (*P*^*αbur*^_*ij*_: 0.97–1.88) is favored in buried regions (Figure 2B) for more than 80% of the folds. Pro and Gly are extremely disfavored in both exposed and buried regions for more than 92% of the folds. The propensities of Ala in the exposed and buried regions of α-helix have a similar tendency as *P*^*α*^_*ij*_. Ala is favored in the α-helical conformation in both exposed and buried regions for 72% and 79% of the folds, respectively, whereas Ala is disfavored by 8% and 13% of the folds when exposed or buried, respectively. For the "4-helical cytokines" fold, the values of the propensity of Ala in both exposed and buried regions are also low (*P*^*αexp*^_*ij*_ = 0.72 and *P*^*αbur*^_*ij*_ = 0.60). A wide range of *P*^*αbur*^_*ij*_ values was obtained for the aromatic residues Phe and Tyr, depending on fold type (Figure 2B), like as *P*^*α*^_*ij*_.

### Fold dependency of amino acid propensities for β-strands

As shown in Figure 1B, a wide range of *P*^*β*^_*ij*_ values was obtained for Trp (0.45–2.22), Thr (0.73–1.87), Lys (0.46–1.45) and Arg (0.51–1.42) depending on fold type. For Lys, although *P*^*β*^_*ij*_ was <0.9 in 18 of 24 folds (mean value of *P*^*β*^_*ij*_ = 0.79), three folds (the lipocalins fold, OB-fold, and protein kinase–like fold) yielded *P*^*β*^_*ij*_ values > 1.2, which had the population differences corresponding to 90% confidence level with that of other folds. These three folds are “all-β” or “α + β”, and all have largely exposed β-strands, whereas β-strands are usually covered by α-helical or loop regions, especially in “α/β” proteins (Table 1). It has long been thought that β-strands prefer hydrophobic residues [1,6,10]; however, it now appears that largely exposed β-sheet structures prefer hydrophilic residues such as Lys. In contrast, the four amino acids Val, Ile, Phe and Tyr are favored (*P*^*β*^_*ij*_ > 1.1) in β-strands of more than 80% of folds, with Val (1.40–2.68) and Ile (1.17–2.33) having particularly high propensities in this regard. The six amino acids Pro, Ala, Asn, Asp, Glu and Gly are disfavored (*P*^*β*^_*ij*_ < 0.9) in β-strands for more than 80% of folds, and Pro (0.16–0.71) and Asp (0.22–0.91) have quite low propensities.

The exposed residue fractions were observed in the range from about 10% to 46% for 24 folds (Table 1) and Glu and Lys have strong and positive correlations between the amino acid propensities and the exposed residue fractions of β-strands in each fold (R_E_ = 0.76, R_K_ = 0.73). Gln, Arg and Ile also have relatively strong correlations, although the correlation for Ile is negative (R_Q_ = 0.67, R_R_ = 0.5, R_I_ = −0.68). As opposed to the strong positive correlation found for Glu, there is no correlation for the other negatively charged amino acid, Asp. The exposed residue fraction appears to be one of the major factors governing charged amino acid composition of folds for β-strands.

For residues exposed in a β-strand (Figure 2C), a wide range of *P*^*βexp*^_*ij*_ values was obtained for Ser (0.42–1.69), Lys (0.84–1.58) and Arg (0.68–1.85). A wide range of *P*^*βbur*^_*ij*_ values was obtained for Cys (0.61–2.61), Phe (0.66–1.83), Tyr (0.64–1.92), Trp (0.31–1.77) and His (0.41–1.87) for residues buried in a β-strand (Figure 2D). *P*^*βexp*^_*ij*_ values of Val, Ile, Phe, Tyr, Trp and Thr are high (*P*^*βexp*^_*ij*_ > 1.1) for more than 75% of folds, indicating that these amino acids, which have a β-branched or aromatic side chain, are favored in the exposed regions of β-strands in all fold types. In contrast, amino acids that are disfavored in all folds in β-strands are Pro (0.22–0.87), Ala (0.28–0.70) and Gly (0.23–0.88) for exposed regions, and Pro (0.12–0.87) for buried regions. It is interesting that *P*^*βexp*^_*ij*_ values for all folds for Ala are lower by comparison (*P*^*βexp*^_*ij*_ < 0.7), indicating that an exposed residue on a β-strand is an extremely unfavorable position for Ala as well as for Pro and Gly. These strong tendencies support that the backbone solvation is a major factor determining thermodynamic β-propensities [32].

### Correlations between amino acid propensities and SCOP fold

**Figure 3:**
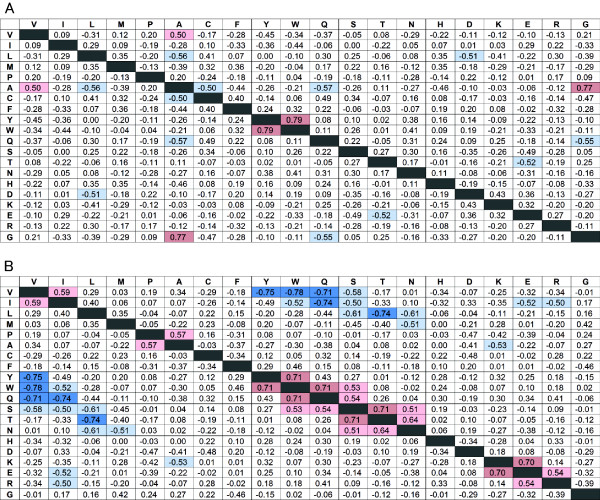
**Correlation coefficients between amino acid propensities. **Correlation coefficients between amino acid propensities for α-helices (**A**) and β-strands (**B**). Strong negative correlations (R < −0.7) are indicated by dark blue, and positive correlations (R > 0.7) are indicated by dark red. Comparatively strong negative correlations (R < −0.5) are indicated by light blue and positive correlations (R > 0.5) by pink.

**Figure 4:**
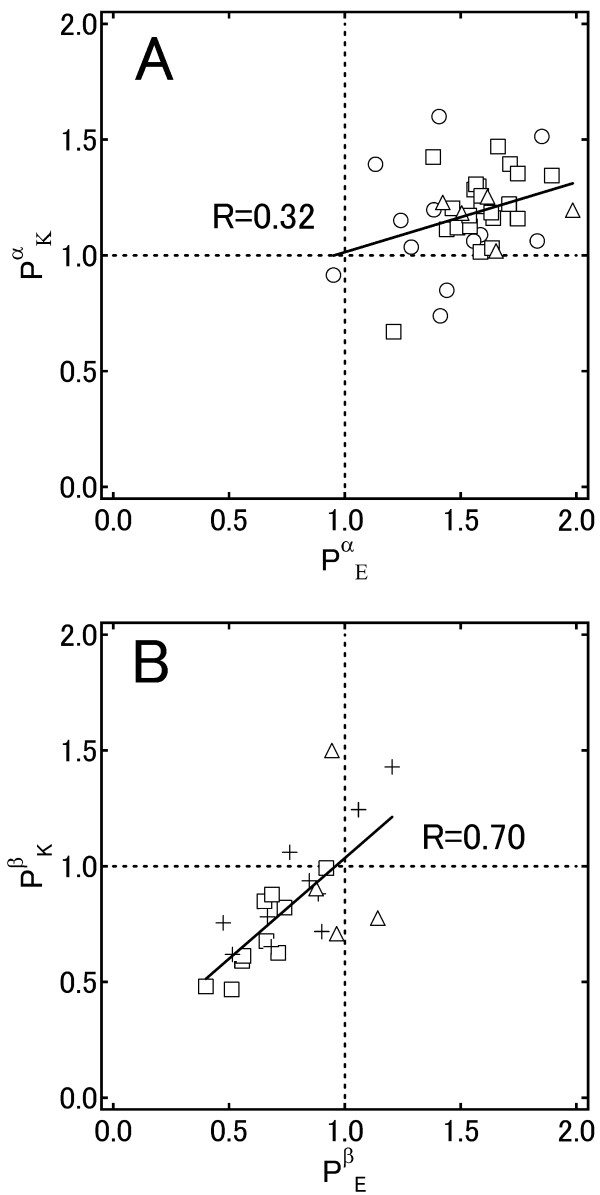
**Relationship between the amino acid propensities. **Amino acid propensities, P, for Glu and Lys for each SCOP fold for α-helices (**A**) and β-strands (**B**). The SCOP classes are: all-α proteins (○), α/β proteins (□), α + β proteins (Δ) and all-β proteins (+).

To investigate the factors that determine the fold dependence of the amino acid propensity for the secondary structures, correlation coefficients were calculated using amino acid propensities obtained from 39 SCOP folds for α-helices (Figure 3A) and 24 SCOP folds for β-strands (Figure 3B). Figure 4, for example, shows the relationships between the propensities of Glu and Lys for α-helices and β-strands. Each data point represents a fold in which more than 2,000 residues are found in each of α-helices and β-strands. For β-strands (Figure 4B), these two amino acid propensities have a correlation coefficient of 0.70, which suggests that folds rich in Glu are likely to also be rich in Lys. In contrast, for α-helices (Figure 4A) no significant correlation was observed. For β-strands, “α/β” proteins (□ in Figure 4B) show low propensities for Glu and Lys, although lipocalins and OB-folds (both “all-β”, + in Figure 4B) show higher propensities for Glu and Lys. For “α+β” proteins (▵ in Figure 4B), there is no correlation between the propensities of Glu and Lys. The correlation coefficients for “all-β” proteins and “α/β” proteins are 0.83 and 0.86, respectively.

Overall, there is a greater number of strong correlations (R < −0.7 or R > 0.7) for β-strands than for α-helices (Figure 3). For example, four strong positive correlations and five strong negative correlations are observed for β-strands, but there are only two paired strong correlations for α-helices (Ala and Gly, Tyr and Trp). Most of the positive correlations for β-strands involve paired amino acids having similar physicochemical characters (shown along the diagonal in Figure 3B), such as Val and Ile, Tyr and Trp, Ser and Gln/Thr/Asn, Asn and Thr, and Glu and Lys/Arg. In contrast, most of the negative correlations for β-strands involve pairs of amino acids having different physicochemical characters, such as Val and Tyr/Trp/Gln/Ser, Ile and Trp/Gln/Ser/Glu/Arg, Leu and Ser/Thr/Asn, Met and Asn, and Ala and Lys.

Interestingly, the aromatic amino acid, Phe, shows low correlations with Trp and Tyr, for both α-helices and β-strands, although strong positive correlations between Trp and Tyr are observed for both α-helices and β-strands.

### Correlations between SCOP fold and propensities for exposed or buried amino acids

**Figure 5:**
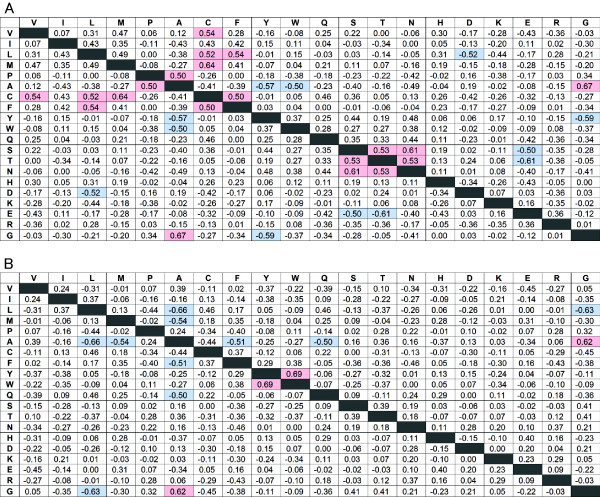
**Correlation coefficients between α-helix propensities for exposed residues and buried residues. ** Correlation coefficients between α-helix propensities for exposed residues (**A**) and buried residues (**B**). Strong negative correlations (R < −0.7) are indicated by dark blue, and positive correlations (R > 0.7) are indicated by dark red. Comparatively strong negative correlations (R < −0.5) are indicated by light blue and positive correlations (R > 0.5) by pink.

**Figure 6:**
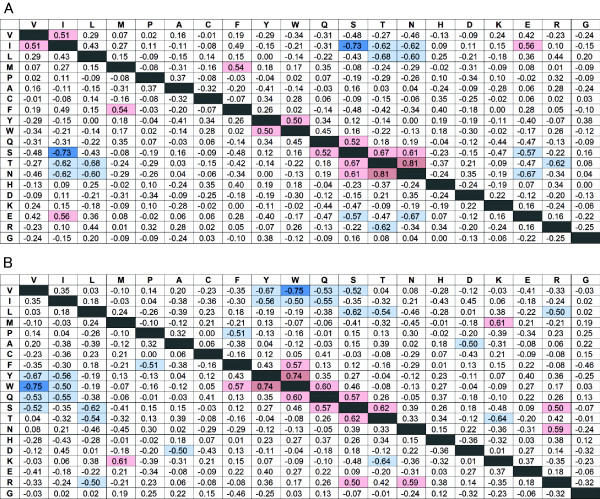
**Correlation coefficients between β-sheet propensities for exposed residues and buried residues. ** Correlation coefficients between β-sheet propensities for exposed residues (**A**) and buried residues (**B**). Strong negative correlations (R < −0.7) are indicated by dark blue, and positive correlations (R > 0.7) are indicated by dark red. Comparatively strong negative correlations (R < −0.5) are indicated by light blue and positive correlations (R > 0.5) by pink.

We also calculated correlation coefficients for amino acid propensities of exposed and buried residues for α-helices (Figure 5), β-strands (Figure 6) and other conformation (Data not shown). Although amino acid propensities for α-helices have two strong correlations (Figure 3A), there is no strong correlation for exposed (Figure 5A) and buried (Figure 5B) residues for α-helices. The strong positive correlation between Trp and Tyr for all residues was absent for exposed residues, but a weak positive correlation was observed for buried residues. These results indicate that a fold that favors Trp on the interior side of an α-helix also favors Tyr in a interior of α-helices. Again, Phe had no correlation with Trp or Tyr for exposed or buried residues. The positive correlations among Ser, Asn and Thr, and the negative correlations between Ser/Thr and Glu, were observed only for exposed residues. Although some new correlations were observed, these values were relatively low for α-helices. For other conformation, strong correlation was not observed for both exposed and buried residues.

### Correlation for buried amino acids in β-strand

In contrast, for β-strands, most of the correlations shown in Figure 3B are strong correlations for exposed (Figure 6A) and buried (Figure 6B) residues. The strong negative correlations for Val/Ile and Tyr/Trp/Gln were observed for buried but not exposed residues. In other words, a fold type that prefers Val or Ile does not prefer Tyr, Trp or Gln, especially for buried residues.

**Figure 7:**
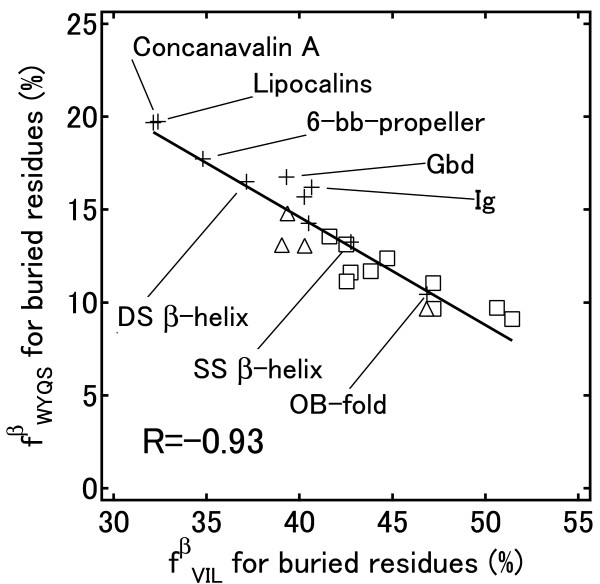
**Relationship between the frequencies of buried residues. **Relationship between the frequencies of buried Val, Ile and Leu residues, *f *_*VIL *_, and buried Trp, Tyr, Gln and Ser residues, *f *_*WYQS *_, in β-strands. The SCOP classes are: α/β proteins (□), α + β proteins (Δ) and all-β proteins (+).

By visually inspecting buried residues for β-strands in the SCOP fold group of “concanavalin A–like lectins/glucanases” (concanavalin A), in addition to buried Tyr and Trp residues we found many polar amino acids such as Gln, Ser or Thr, and charged amino acids such as Glu, Lys or Arg, involved in H-bonds with each other to counterbalance the polarity in the hydrophobic environment. For the buried residues, we calculated the correlation coefficients between the combined frequencies of hydrophobic amino acids (Val, Ile and Leu) and some polar amino acids (Table 3 and Figure 7). The correlation coefficients calculated from the frequencies are the same as those calculated from the propensities, and thus it is easier to understand the amino acid occurrences. The combined frequencies of Trp, Tyr and Gln that are buried have a strong correlation (R = −0.87) with those of hydrophobic amino acids (Val, Ile and Leu). The inclusion of Ser in the group with Trp, Tyr and Gln increased the correlation coefficient to −0.93 (Figure 7). The fact that the correlation coefficients for Val/Ile/Leu and Tyr/Trp/Gln/Ser range from −0.19 to −0.75 indicates synergy in the correlation of the combined frequencies for β-strands that does not exist for α-helices and other conformation (Table 3). The synergy between these amino acid groups suggests that the amino acids within the same group can be exchanged. For example, in a fold type where Leu is preferred for buried residues, Ile will also be preferred. Thus, at buried sites, fold types with many aliphatic residues (Val, Ile and Leu) also contain low quantities of Tyr, Trp, Gln and Ser. Figure 7 also shows that “all-β” proteins tend to have a higher content of Tyr, Trp, Gln and Ser, whereas “α/β” proteins have a higher content of aliphatic amino acids at buried sites. The top six folds for the content of Tyr, Trp, Gln and Ser at buried sites in β-strands are “all-β” proteins and have two large β-sheets packed together (lipocalins, concanavalin A, 6-bladed beta-propeller (6-bb-propeller), galactose-binding domain-like (Gbd), double-stranded β-helix (DS β-helix), and immunoglobulin-like beta-sandwich folds (Ig)). Other “all-β” proteins that consisted of only one small β-sheet or small β-barrel structure have a small hydrophobic core. The H-bonds between the buried side chains may be necessary for correct alignment of two large β sheets in particular.

**Table 3:** Correlation coefficients for buried residues

	**α-helix**	**β-strand**	**Other**
*f *_*WYQ *_ vs. *f *_*VI*_	−0.51	−0.87	−0.24
*f *_*WYQ *_ vs. *f *_*VIL*_	−0.22	−0.87	−0.26
*f *_*WYQS *_ vs. *f *_*VIL*_	−0.31	−0.93	−0.52

### Correlation for exposed amino acids in β-strand

Negative correlations for Ile/Leu and Ser/Thr/Asn were observed in the exposed residues (Figure 6A), although the correlations for Ile and Thr/Asn were not observed when both exposed and buried residues were calculated together (Figure 3B). Negative correlations were also observed for Glu and Ser/Asn and for Arg and Thr. We examined the correlation of the combined frequencies for these exposed amino acids in β-strands as shown in Table 4. This result shows that strong correlations exist in the frequencies of certain hydrophobic amino acids (Ile, Leu), charged amino acids (Glu, Lys, Arg), and polar amino acids (Ser, Thr, Asn) in the exposed regions of β-strands. It is interesting that the frequencies of hydrophobic (Ile, Leu) and charged (Glu, Lys, Arg) amino acids correlate negatively with those for polar amino acids (Ser, Thr, Asn). A common feature for Ile, Leu, Glu, Lys and Arg is that they have relatively long side chains, including more than two hydrophobic methylene groups, whereas Ser, Thr and Asn have short side chains.

**Table 4:** Correlation coefficients for solvent-exposed residues

	**α-helix**	**β-strand**	**Other**
*f *_*IL *_ vs. *f *_*STN*_	−0.21	−0.79	−0.51
*f *_*EKR *_ vs. *f *_*STN*_	−0.57	−0.76	−0.61
*f *_*ILEKR *_ vs. *f *_*STN*_	−0.59	−0.90	−0.66

**Figure 8:**
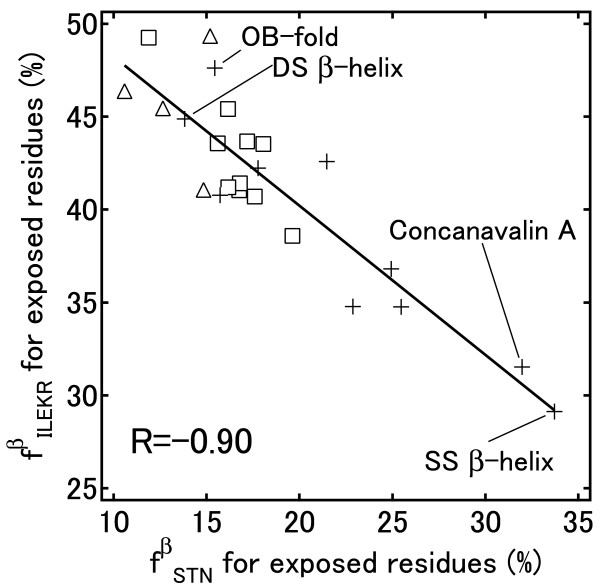
**Relationship between the frequencies of exposed residues. **Relationship between the frequencies of exposed Ile, Leu, Glu, Lys and Arg residues, *f *_*ILEKR *_, and exposed Ser, Thr and Asn residues, *f *_*STN *_, in β-strands. The SCOP classes are: α/β proteins (□), α + β proteins (Δ) and all-β proteins (+).

**Figure 9:**
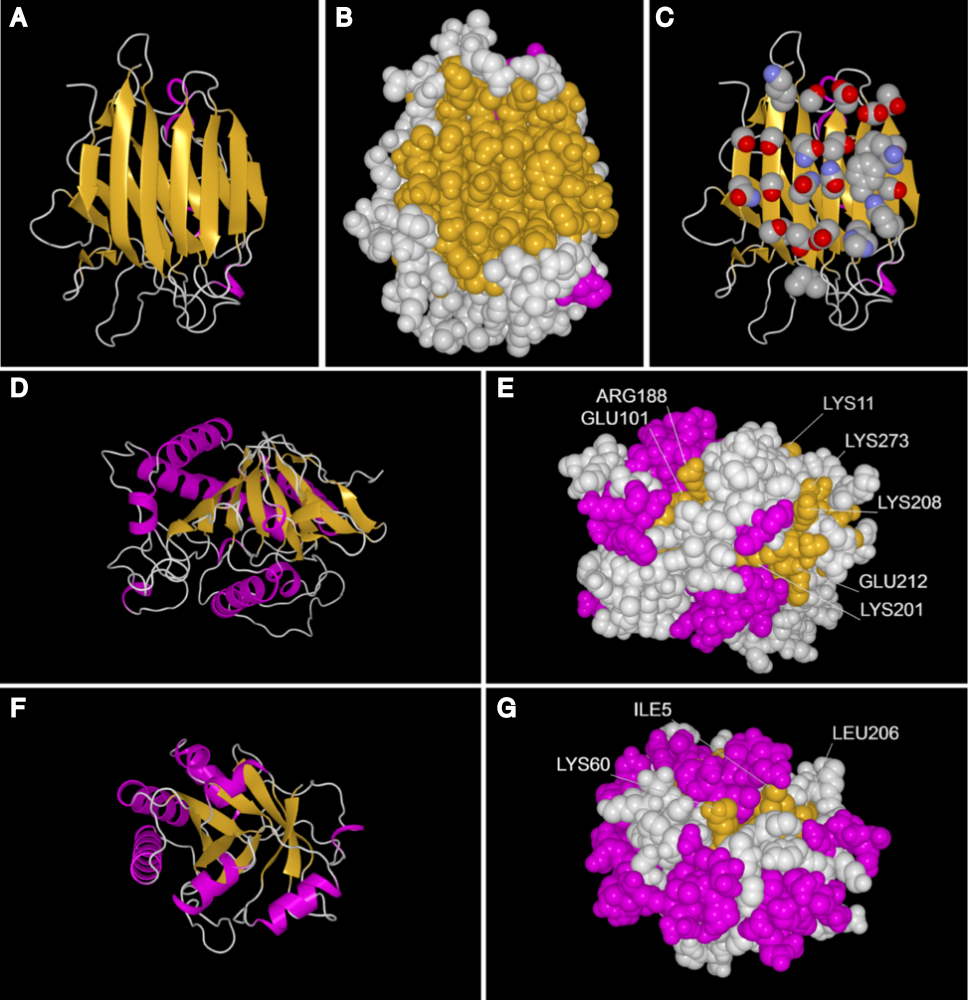
**Amino acid residues on β-strands of three folds. **Amino acid residues in β-strands of concanavalin A (**A**, **B** and **C**, PDB ID:1IOA), DS β-helix (**D** and **E**, PDB ID:1ODM), and TIM barrel (**F** and **G**, PDB ID:1SFS). The residues for α-helices are colored magenta, and those for β-strands are colored yellow. The side chains of residues in β-strands are colored by atom type (nitrogen: blue, oxygen: red, carbon: grey) in C.

Figure 8 shows a strong correlation between the combined groupings of Ser, Thr and Asn with Ile, Leu, Glu, Lys and Arg (R = −0.90). For the exposed regions of β-strands, it is clear that in all “α/β” proteins and all “α+β” proteins, Ile, Leu, Glu, Lys and Arg are preferred and that Ser, Thr and Asn are disfavored. Fold types that prefer Ser, Thr or Asn have a relatively low content of Ile, Leu, Glu, Lys, or Arg, and they are “all-β” proteins. Figure 8 also shows the widespread distribution of the folds of “all-β” proteins. For the two SCOP folds DS β-helix and OB-fold of “all-β” proteins, the residues Ile, Leu, Glu, Lys or Arg are preferred in the exposed regions of the β-strands. These fold types have twisted and bent β-strands. Some C_α_ atoms in the β-strands are positioned at the bottom of the narrow and deep valley formed by the twisted and bent β-strands (Figure 9D and E). At such positions, the short, polar side chain of Ser, Thr or Asn is unable to reach the solvent, so amino acids with long side chains are favored. Much the same is true for “α/β” proteins (Figure 9F and G). The β-sheet is covered by α-helices and twists in “α/β” proteins, leaving only narrow spaces for the residues at the ends of the β-strands to reach solvent. In contrast, the two SCOP folds concanavalin A and single-stranded right-handed β-helix (SS β-helix) have a remarkably high content of Ser, Thr and Asn in the exposed regions of β-strands and have largely exposed and flat β-sheets (Figure 9A, B and C). Figure 9C shows that Ser, Asn and Thr are dominant in the flat β-sheet, and they do not significantly make contact with each other. These results suggest that amino acid composition in the exposed regions of β-strands governs the formation of a twist in β-sheets.

Wang et al. [33] showed that isolated β-strands in molecular dynamics simulations are not twisted, suggesting that the stabilization of the twist must be due to inter-strand interactions. Another computer simulation study found that inter-strand interactions by side chains induce a twist and that β-branched side chains are important for twist formation [34]. On the other hand, Koh et al. [35] and Bosco et al. [36] used statistical analyses to show that β-sheet structure is mainly determined by the backbone, and the contribution of side chains is small. This indicates that twisting is an inherent property of a polypeptide chain, implying that a β-strand should twist regardless of its amino acid sequence. However, some folds have a large/flat β-sheet, such as the SCOP groups concanavalin A and SS β-helix. Previous studies have targeted only the twisted β-strand and not focused on the flat β-sheet. Our results suggest that the amino acid composition in the exposed regions of β-strands may be related to the twist and bend of the strand, showing that side chain interactions are also an important factor for β-strand twisting. An intuitive explanation is that the long side chains of Leu, Ile, Lys, Arg and Glu in the exposed regions come close together to form the hydrophobic core, resulting in the formation of a twist and/or bend in β-strands. In contrast, the side chains of Ser, Thr and Asn have low hydrophobicities and are short so that the hydrophobic interactions between the side chains are weak and produce a flat β-sheet. Therefore, it seems that the strain within a β-sheet is one of the major factors governing amino acid propensities of folds for β-strands.

### The types of β-sheets and the amino acid propensity

The folds can be classified by their β-sheet types into three; parallel, antiparallel and mixed β-sheet. For "all-β" protein class and "α + β" protein class, β-sheets of all folds used in this study are completely antiparallel β-sheet except for SS β-helix which has completely parallel β-sheet. The folds of "α/β" protein class have completely or mainly parallel β-sheets. β-sheets of the three folds, "Flavodoxin-like", "NAD(P)-binding Rossmann-fold domains" and "TIM beta/alpha-barrel" are completely parallel, whereas "Periplasmic binding protein-like II" and "Thioredoxin fold" have mixed β-sheet.

For the exposed residues of β-strands (Figure 8), the plots for the folds of "all-β" proteins class were widely distributed, although they are commonly completely antiparallel β-sheet except for SS β-helix. Furthermore, the folds of "α/β" proteins class have different amino acid compositions from that of SS β-helix, although they have parallel β-sheets. Figure 7 shows that the plots for the folds of "all-β" proteins class were widely distributed and the plot of SS β-helix is in the center of the graph. The residue fractions (*f*^*βbur*^_*VIL*_) of the three folds that have completely parallel β-sheets were also widely distributed (51.4, 47.2 and 42.7%).

These results indicate that the correlations found in Figure 7 and 8 cannot be explained by the types of β-sheets. Consequently, we think that the propensities do not depend on the types of β-sheets.

### Robustness of the dataset

We checked the robustness of our results using the dataset of more than 1,500 residues and less than 2,000 residues, which is not included in the dataset used in this study; six folds for α-helix and eight folds for β-strands. For β-strands, strong correlations were also observed for buried residues (R_WYQS-VIL_ = −0.81) and for exposed residues (R_ILEKR-STN_ = −0.78). There are no strong correlations for buried residues (R_WYQS-VIL_ = −0.64) and for exposed residues (R_ILEKR-STN_ = −0.48) in α-helices. These results are the same as those obtained for the dataset containing more than 2,000 residues. Therefore, the results presented here seem to be independent of the dataset selection.

## Conclusion

The amino acid propensities for secondary structures were investigated for each SCOP fold. The helix propensities calculated for exposed and buried residues are also similar to each other. For β-sheet propensities, however, propensities calculated for exposed residues are remarkably different from those of buried residues, which are similar to those calculated for all residues because β-sheets tend to be located in the interior of proteins.

We also detected correlations between amino acid compositions in β-strands. At buried sites, the content of Tyr, Trp, Gln and Ser correlates negatively with the content of the aliphatic amino acids Val, Ile and Leu. All-β proteins tend to have a higher content of Tyr, Trp, Gln and Ser, whereas α/β proteins tend to have a higher content of aliphatic amino acids at buried sites. In all-β proteins, the H-bonds between buried side chains may be necessary for correct alignment of two large β sheets. For exposed residues, there is a tendency that a fold with a high content of Ile, Leu, Glu, Lys and Arg would have a low content of Ser, Thr and Asn. Generally, α/β proteins have twisted and bent β-strands and favor longer side chains at exposed sites.

These findings are very useful for the design of β-sheet. They are especially effective when there is structural information such as whether a residue is exposed or buried, two large β-sheets are packed together, a β-sheet has α-helices at least one side of β-sheets and a β-strand is twisted or not. Hecht and coworkers have succeeded in designing de novo proteins with binary patterning techniques, in which polar and non-polar amino acids are placed at desired sites along the sequence by synthesizing DNA with degenerated codon [37]. If one desire to design a de novo protein library of SS β-helix, for example, he should consider to bias in favor of Ser, Thr, and Asn rather than Glu, Lys, Arg for exposed sites on β-strands because the frequency of Ser, Thr, and Asn is relatively high and conversely the frequency of Ile, Leu, Glu, Lys, Arg is low for exposed sites on β-strands of SS β-helix folds (Figure 8).

## Abbreviations

R: Correlation coefficient.

## Competing interests

The authors declare that they have no competing interests.

## Authors’ contributions

KF conceived the project and wrote the manuscript. HT wrote the programs and performed all analyses. MI participated the discussion of the project and was involved the revision of the manuscript. All authors read and approved the final manuscript.
